# Characterization of the High-Affinity Drug Ligand Binding Site of Mouse Recombinant TSPO

**DOI:** 10.3390/ijms20061444

**Published:** 2019-03-21

**Authors:** Soria Iatmanen-Harbi, lucile Senicourt, Vassilios Papadopoulos, Olivier Lequin, Jean-Jacques Lacapere

**Affiliations:** 1 Sorbonne Université, Ecole Normale Supérieure, PSL University, CNRS, Laboratoire des Biomolécules (LBM), 4 place Jussieu, F-75005 Paris, France; soriatmanen@hotmail.com (S.I.-H.); lucile.senicourt@gmail.com (I.S.); olivier.lequin@upmc.fr (O.L.); 2 Department of Pharmacology and Pharmaceutical Sciences, School of Pharmacy, University of Southern California, Los Angeles, CA 90089, USA; vpapadop@usc.edu

**Keywords:** translocator protein (TSPO), ligand binding site, nuclear magnetic resonance (NMR), trypsin digestion, circular dichroism (CD), intrinsic fluorescence

## Abstract

The optimization of translocator protein (TSPO) ligands for Positron Emission Tomography as well as for the modulation of neurosteroids is a critical necessity for the development of TSPO-based diagnostics and therapeutics of neuropsychiatrics and neurodegenerative disorders. Structural hints on the interaction site and ligand binding mechanism are essential for the development of efficient TSPO ligands. Recently published atomic structures of recombinant mammalian and bacterial TSPO1, bound with either the high-affinity drug ligand PK 11195 or protoporphyrin IX, have revealed the membrane protein topology and the ligand binding pocket. The ligand is surrounded by amino acids from the five transmembrane helices as well as the cytosolic loops. However, the precise mechanism of ligand binding remains unknown. Previous biochemical studies had suggested that ligand selectivity and binding was governed by these loops. We performed site-directed mutagenesis to further test this hypothesis and measured the binding affinities. We show that aromatic residues (Y34 and F100) from the cytosolic loops contribute to PK 11195 access to its binding site. Limited proteolytic digestion, circular dichroism and solution two-dimensional (2-D) NMR using selective amino acid labelling provide information on the intramolecular flexibility and conformational changes in the TSPO structure upon PK 11195 binding. We also discuss the differences in the PK 11195 binding affinities and the primary structure between TSPO (TSPO1) and its paralogous gene product TSPO2.

## 1. Introduction

The 18 kDa translocator protein (TSPO), previously named PBR for peripheral-type benzodiazepine receptor [1], is an evolutionarily conserved membrane protein [2] located in eukaryotic cell mitochondria. TSPO is highly expressed in steroidogenic and bile salt-synthesizing cells [1,3], but it has been also observed in many other tissues [1]. Despite its implication in many cellular functions and the high number of pharmacological studies, little is known about its structure–function relationships that may limit its pharmacological efficiency. However, TSPO overexpression in neuroinflammation and neurodegenerative disorders has made TSPO and its possible ligands extremely attractive subcellular targets for diagnostics and therapeutics [4,5,6]. 

Many TSPO ligands belonging to different chemical classes have been identified over the last decades [4], but overly complex binding profiles likely due to the number of genetic variants [7] as well as the lack of atomic structures have not permitted the optimization of drug design [8]. Recently, the atomic structure of recombinant mouse TSPO (rec-mTSPO) was determined by NMR [9] (PDB ID-2MGY), after stabilization by its high-affinity drug ligand, PK 11195 [10]. Previous studies sought to understand the origin of the selectivity of TSPO toward PK 11195. Various mutations and deletions have been reported in mammalian and bacterial species [11,12,13,14], suggesting the involvement of the five transmembrane helices and the cytosolic loops, which were then confirmed by the atomic structures determined for mammalian and bacterial TSPO [9,15,16]. The ligand binding pocket that has been characterized in these atomic structures reveals the involvement of several conserved residues [9,15,16]. Some are rigorously identical among the different species of TSPO isoform 1, but others are just homologous. The most significant amino acid differences are observed when comparing TSPO (TSPO1) to its paralogous gene product TSPO2 that correlates with the observed differences in the PK 11195 binding affinities for these proteins [17].

The precise mechanism of ligand access to its pocket from the cytosol remains unknown. The crystal structures of bacterial TSPO (*Bc*TSPO and *Rs*TSPO for *Bacillus cereus* and *Rhodobacter sphaeroides*, respectively) with and without ligands give highly superimposable atomic structures with cytosolic loops closing the entrance to the binding site [15,16], giving no indication of the dynamic changes needed to understand ligand binding. The NMR structure of the A147T polymorph of mammalian TSPO, described as decreasing the binding affinity for PK 11195 [7], exhibits a very similar structure to the wild type (WT) with ligand bound [18], as measured by a root-mean-square deviation (CA-RMSD) of 1.3 Å for all carbon alpha atoms. A147T substitution modulates the structure around the site mutation but also induces local rearrangements of the cytosolic domains and, in particular, the cytosolic end of the first transmembrane helix [18]. A larger CA-RMSD of 3.0 Å can be measured for the TM1-TM2 connecting loops (E29-R46) for A147T compared to WT protein, suggesting that this part might be important for the access of PK 11195 to the binding pocket [18]. Interestingly, in the A147T mutant, residue Y34 is oriented toward the binding site and is facing the aliphatic moiety of PK 11195, whereas it is oriented outward in the WT protein (corresponding to a change in the chi1 rotamer). The dynamics of this TSPO region and of Y34, in particular, suggest a contribution of this residue to the accessibility of the binding site. 

Interestingly, a large fluctuation of the structure is also observed between mTSPO and *Bc*TSPO with bound PK 11195 for the segment connecting TM3 and TM4 and comprising a highly conserved F100. The side chain of this residue is solvent-exposed in the WT-mTSPO but is oriented toward the PK 11195 binding site at 4 Å in *Bc*TSPO [16], suggesting a possible contribution of this residue in the accessibility of the ligand to the pocket and/or the positioning of the ligand within its binding site. 

Deletion mutants previously described [13,14] are interesting to overview (Figure S1) because they concern large protein fraction deletions and they could strongly affect protein folding, specifically within the TM helices and, thus, ligand binding. However, among the various deletion mutants described, only the Δ15–35 mutant of human TSPO, i.e., involving a large part of TM1, loses its binding capacity when overexpressed in yeast. Interestingly, two deletion mutants in the cytosolic part of the TSPO (Δ41–51 and Δ153–169) show reduced binding properties (55% and 75% of the control, respectively). The first one (Δ41–51) involves several residues from the TM2 involved in the binding of PK 11195 within the atomic structure. It also lacks several highly conserved residues, W42, P44 and P45, that are not part of the TM2 but might contribute to the access of the binding site. The second (Δ153–169) is the C-terminus domain where the first residues (153–159) belong to TM5 and makes several contacts with the TM1-TM2 and TM3-TM4 loops [18]. Such a deletion might destabilize the folding and stability and, thus, alter the affinity and stoichiometry of binding.

In this report, we have repeated the studies illustrating mTSPO1 structural changes upon ligand binding using circular dichroism and quantify the binding of PK 11195 to the protein in detergent. We have specifically labelled the two unique lysine residues located on opposite sides of the protein and recorded their NMR spectra in the presence and absence of the ligand, revealing the large conformational change. We have designed limited proteolysis studies to show changes in the accessibility to trypsin upon ligand binding. We have focused our mutagenesis study on conserved aromatic amino acids residues (Y34 and F100) located in the cytoplasmic loops that could be involved in the access of the ligand to its binding pocket. We have also included two deletion mutants of cytosolic facing domains (Δ41–51 and Δ153–169) to compare their contribution to the point mutations. We expressed these point mutants, as well as deletion mutants, investigated their secondary structure and characterized the effects of the mutations on PK 11195 binding. We also discussed the global conformation change of TSPO upon ligand binding, the specific involvement of the cytoplasmic loops and the differences in amino acid sequences between TSPO1 and TSPO2 proteins that may account for the ability of TSPO1 to bind drug ligands.

## 2. Results

**Figure 1:**
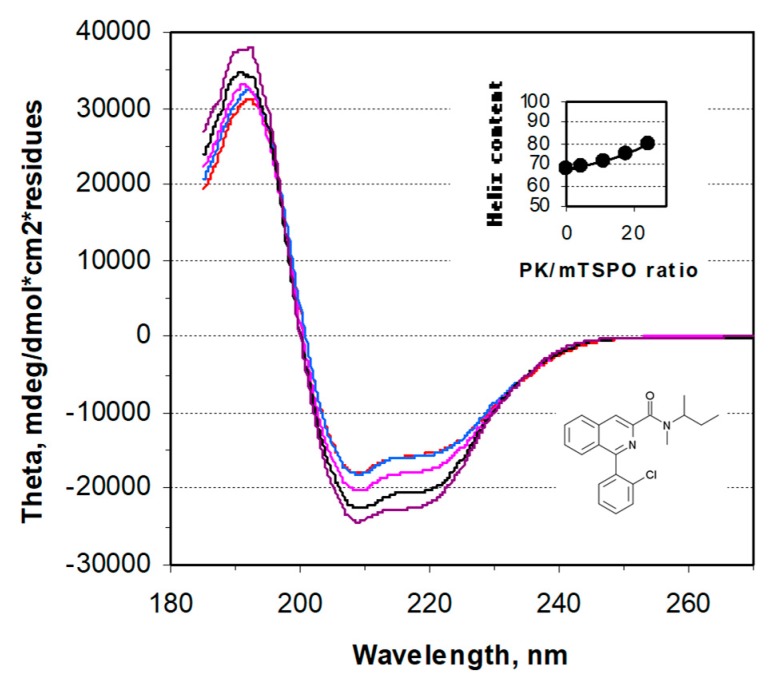
The circular dichroism spectra of rec-mTSPO: The spectra recorded in the presence of dodecylphosphocholine (DPC) detergent and increasing amounts of PK 11195 (its structure is given in the bottom right of the panel). The insert shows the increase in the total helix content of rec-mTSPO upon PK 11195 addition, expressed as a molar ratio over protein.

The overexpression of rec-mTSPO in heterologous cells permits the production of large amounts of protein by extraction from bacterial inclusion bodies using sodium dodecyl sulfate (SDS) as detergent [19]. SDS-solubilized rec-mTSPO showed partial helical folding as previously described and shown in the circular dichroism (CD) spectrum [19] (Figure S2). However, no PK 11195 binding was observed in the presence of this detergent [19]. Exchanging the SDS detergent for dodecylphosphocholine (DPC) increased the helix folding percentage of rec-mTSPO as previously described and inferred from the change in CD spectrum [19] (Figure S2). PK 11195 binding to rec-mTSPO was observed in the presence of DPC [10]. The addition of increasing amounts of PK 11195 induced increasing changes in the CD spectrum, as shown in Figure 1, but required high ligand over protein molar ratios (inset of Figure 1), suggesting a low affinity compared to the native membrane [12]. This ratio does not reflect just binding, since part of the PK 11195 is bound into the DPC micelles, reducing the free PK 11195 concentration available for the protein [10]. 

**Figure 2:**
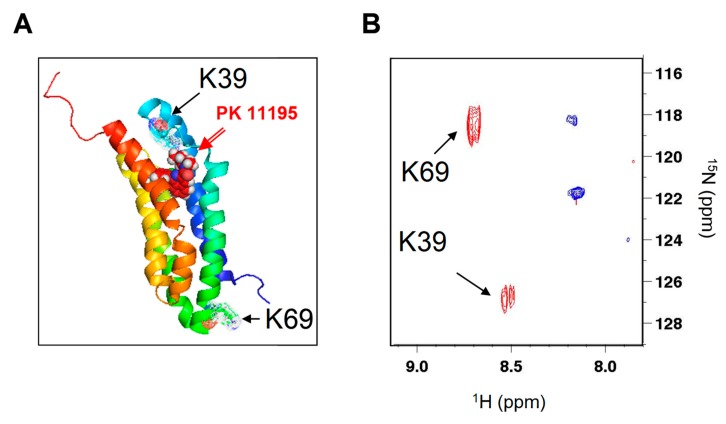
The ligand-induced stabilization of the rec-mTSPO structure in dodecylphosphocholine (DPC) detergent: (**A**) the atomic NMR structure of rec-mTSPO with PK 11195 bound [9] (PDB ID-2MGY) emphasizing the positions of the two lysines (K39 and K69) and (**B**) the 2-D ^1^H–^15^N HSQC spectra of selectively [^15^N]-Lys labelled rec-mTSPO in the absence (blue) and in the presence (red) of PK 11195.

NMR studies previously showed that ligand binding stabilizes TSPO tertiary folding, [9,10,19] as revealed in the 1-D ^1^H NMR spectrum with the presence of several upfield shifted methyl resonances as described [10,19]. In the 2-D ^1^H–^15^N HSQC spectra, the addition of excess PK 11195 induces a large spectral dispersion of the amide signals, indicating conformational changes from the partially folded to folded structure in agreement with previously described CD experiments [10,18]. The NH indole resonances of tryptophan (boxes in Figure S3A,B) were particularly sensitive to PK 11195 binding, since a broad massif was observed in the absence of ligand (Figure S3A), whereas several well-dispersed peaks were detected in its presence (Figure S3B) as previously described [10,19]. The line widths and low chemical shift dispersion of tryptophan NH indole resonances in the absence of ligand suggests the presence of conformational exchange and the absence of a stable tertiary structure [10,19]. The atomic structure obtained by NMR [9] reveals that the two unique lysine residues (among the 189 amino acids of the rec-mTSPO) are located at two opposite sides of the protein (Figure 2A). Therefore, to further study the rec-mTSPO structural changes, we performed selective amino acid labelling and recorded the 2-D ^1^H–^15^N NMR HSQC spectra. Selective [^15^N]-Lys labelling provided an ^1^H–^15^N HSQC spectrum with two peaks, as expected from the amino acid sequence (Figure 2B, blue). The two well-separated peaks were strongly shifted upon PK 11195 binding (Figure 2B, red), suggesting that the lysines are either directly involved in PK 11195 binding or lie in regions affected by PK 11195-induced conformational changes. The first lysine (K39) is in a flexible loop and caps the PK 11195 in its binding site (Figure 2A). The second lysine (K69) is located at the opposite side of the membrane and, thus, revealed a long-range conformational change in this region upon PK 11195 binding. The peaks corresponding to K39 and K69 were detected both in the presence and absence of the PK 11195 ligand, suggesting the absence of conformational heterogeneous states in the vicinity of these residues.

**Figure 3:**
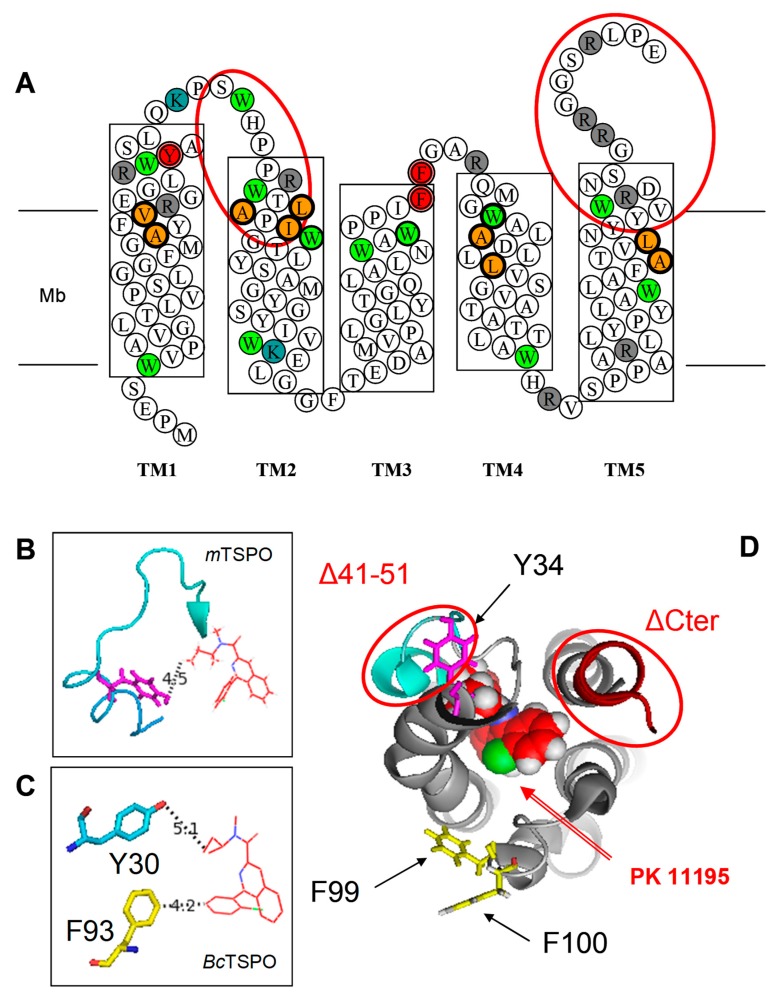
(**A**) The mTSPO sequence in a 2-D diagram with transmembrane helices shown as boxes crossing the membrane (Mb): The point mutations and deletion mutants used in the present work are shown as red-coloured double circles or ellipses, respectively. The amino acids involved in the binding pocket of the atomic structure are shown in orange-filled bold circles. Lysine (K), arginine (R) and tryptophan (W) are shown in blue-, grey- and green-filled circles, respectively. (**B**) The position of Y34 in the NMR structure of A147T polymorph of mammalian TSPO [18] (PDB ID-2NO2) and (**C**) the position of Y30 and F93 in the X-ray structure of bacterial TSPO [16] (PDB ID-4RYI): These two residues are homologous to positions Y32 and F100 of mammalian TSPO. (**D**) The top view of atomic NMR structure with bound PK 11195 [9] (PDB ID-2MGY) emphasizes the positions of the mutations and deletions.

Figure 3 shows that not only lysines but also arginines are located on both sides of the mTSPO. Both types of residues are selectively digested by trypsin; thus, we took advantage of this to perform a limited proteolysis digestion that has been previously used to probe the conformational changes of proteins [20,21]. 

**Figure 4:**
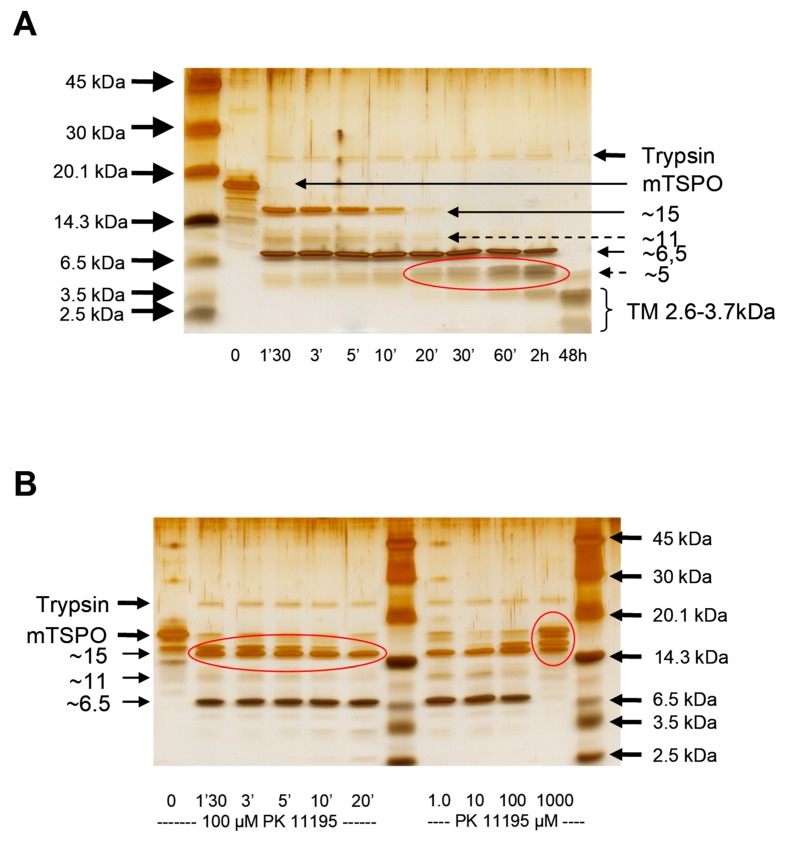
The limited trypsin digestion of rec-mTSPO: (**A**) The time course of the proteolytic cleavage of rec-mTSPO solubilized in DPC by trypsin. In the first minutes of trypsin digestion, two main fragments around 15 and 6.5 kDa were observed. The larger one disappeared over time, whereas shorter peptides below 6.5 kDa started to appear. After 48 h of digestion, only very short peptides around 2.6–3.7 kDa, probably corresponding to the transmembrane TM helices, were visible. (**B**) The effect of PK 11195 upon proteolytic cleavage: the time course in the presence of 100 µM PK 11195 (left panel) and the effect of increasing the concentration of PK 11195 with 3 min of trypsin digestion (right panel): The regions highlighted in red (in panel (**A**)) correspond to the fragment at 5 kDa that is not generated in the presence of PK 11195, whereas the fragments around 15 kDa (highlighted in red in left panel in (**B**)) remain. Moreover, only large fragments are observed in the presence of high concentrations of PK 11195 (red circle in right panel in (**B**)).

The treatment of detergent-solubilized rec-mTSPO with trypsin produced protein fragments which the composition of changed with time (Figure 4A). The presence of PK 11195 protected against cleavage by slowing down the kinetics and led to a different pattern of protein fragments (Figure 4B, left panel). A higher PK 11195 concentration showed increased protection against cleavage for the same incubation time (Figure 4B, right panel). Interestingly, at a high PK 11195 concentration (1 mM), part of the protein was not cleaved, and only large protein fragments were observed. This suggests the existence of conformational changes induced by ligand binding to its pocket that very likely reduces trypsin access to lysines and arginines. It has to be noted that most of the arginines are part of the cytosolic loops and the C-terminal region of the mTSPO, confirming the involvement of these loops in the ligand binding process.

To further study the mechanism of PK 11195 ligand binding, we decided to mutate amino acids and to measure the binding affinities of the mutants. Figure 3 displays the location of the mTSPO amino acids involved in the binding pocket (2-D diagram, top panel). In determining the mutation strategy, we took into consideration (i) our limited proteolysis data, (ii) previous reports of mutagenesis, and (iii) the possible hydrophobic interactions between PK 11195 and aromatic residues. Not all trypsin cleavage sites are immediately accessible to cleavage. Some of them are located close to the PK 11195 binding site, such as R27, R32, K39, and R46, which are in the region connecting TM1 and TM2, as well as R103, which is in the region connecting TM3 and TM4. Our selective labeling NMR spectra confirmed the involvement in ligand interactions of the small helical loop containing K39. The large chemical shift of K39 upon PK 11195 binding supports a strong environment change induced by the ligand. Moreover, based on the available TSPO structural information, with and without ligand [22], together with the hindered location of the binding pocket, we can speculate on a potential conformational change involving hydrophobic interactions between PK 11195 and the aromatic residues. In line with this, several residues are highly conserved in these regions, among which are the W33 and Y34 parts of the end of TM1. A comparison of the NMR structures of WT and A147T mutant showed that Y34 has two distinct orientations (toward and opposite of ligand, Figure 3), whereas W33 only has one, suggesting a potential contribution of Y34 to the PK 11195 binding process and conformational change. Thus, we mutated the highly conserved tyrosine Y34 into either serine or phenylalanine to maintain the hydroxyl or phenyl group of the tyrosine, respectively. This was our first mutant. Our second mutant was the ∆41–51, a large fragment at the end of the connecting region between TM1 and TM2, previously described as having reduced binding properties [14] and containing two conserved tryptophans (W42 and W47) and several other residues as part of the binding pocket (Figure 3A). The third mutant targeted a highly conserved aromatic residue F100 from the short loop connecting TM3 and TM4 that exhibited two different orientations toward the ligand when comparing the mTSPO and *Bc*TSPO structures (Figure 3), which was mutated into alanine, a short chain residue. Our fourth mutant was the ∆153–169, a large fragment at the C-terminus of the protein (Figure 3A), previously described as having reduced binding properties [14] that may stabilize the global 3-D structure of the TSPO by interaction with the TM1-TM2 and TM3-TM4 loops [18]. It contains the cholesterol recognition/interaction amino acids consensus (CRAC) pattern, i.e. the cholesterol binding site [13], and atomic NMR structure shows close interactions between Y153 and Q38S (TM1) on one side and between W155 and M105 (TM4) on the other side that might stabilize folding and stability. Thus, its deletion might change the affinity and stoichiometry of binding by destabilisation. We also constructed double aromatic mutants involving the two loops to test if there may be a “sandwich effect” that may drive PK 11195 to its binding site.

**Figure 5:**
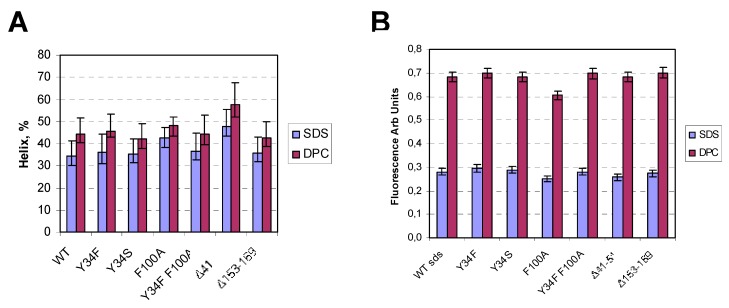
The circular dichroism (CD) and fluorescence studies of rec-mTSPO mutants: the CD-inferred alpha-helix percentage (**A**) and the fluorescence emission intensity (**B**) are shown for the wild type (WT) as well as the mutants in the presence of sodium dodecyl sulfate (SDS) and DPC (blue and red bars, respectively).

In order to check if the mutations and, in particular, the deletions had an effect on the folding of rec-mTSPO, we decided to characterize the secondary structure by recording the circular dichroism spectra (Figure 5A) of the following point mutations and deletion mutants: Y34S, Y34F, F100A, Y34F/F100A, Y34F/F99A, Δ41–51 and Δ153–169. First, these mutants were expressed in *E. coli* and purified in SDS [13,19,23,24]. All mutants seemed folded since they exhibited at least a helical content of around 35%, except mutants F100A and Δ41–51 which showed a level of helicity above 40%. We previously showed (Figure S2) that rec-mTSPO had a higher level of helical content when SDS was exchanged for DPC [19,25]. Therefore, we also recorded the circular dichroism spectra of each mutant in DPC (Figure 5A). We observed an increase in the helical content close to 10% compared to SDS, but some mutants such as Y34S and F100A showed smaller changes, indicating that aromatic interactions might be involved in the folding process. The deletion mutant Δ41–51 that exhibited the highest helical content in SDS (close to 50%) showed an even higher helical content in DPC (close to 60%). Interestingly, both deletion mutants showed a high secondary structure content even when ten to fifteen residues were removed.

The wild type (WT) TSPO has 12 tryptophans, and the intrinsic fluorescence can easily be recorded. We previously showed that the fluorescence intensity is highly dependent on the detergent environment, with large increases when SDS was replaced by DPC [19]. Such increases reflect a change in the tryptophan environment and/or conformational change. We measured the fluorescence intensity of each mutant in the presence of both SDS and DPC and observed a large increase (more than a factor of 2) for all mutants (Figure 5B). Interestingly, Δ41–51 that exhibits the largest helicity change still shows a large fluorescence change, although 2 tryptophans have been deleted. Former studies reported the binding affinity measurements of the ligand to bacterial TSPO [26,27] using intrinsic fluorescence changes. However, these previous reports showed that the addition of PK 11195 to WT TSPO induced a 100% decrease of fluorescence, which does not seem to be a measure of binding but rather a full quenching of the intrinsic fluorescence indicative of more nonspecific interactions. The measurement of “true” binding affinities is not really possible with detergent-solubilized rec-mTSPO, as it usually gives affinities in the micro-millimolar range, probably due to an additional interaction between PK 11195 and detergent [10].

**Figure 6:**
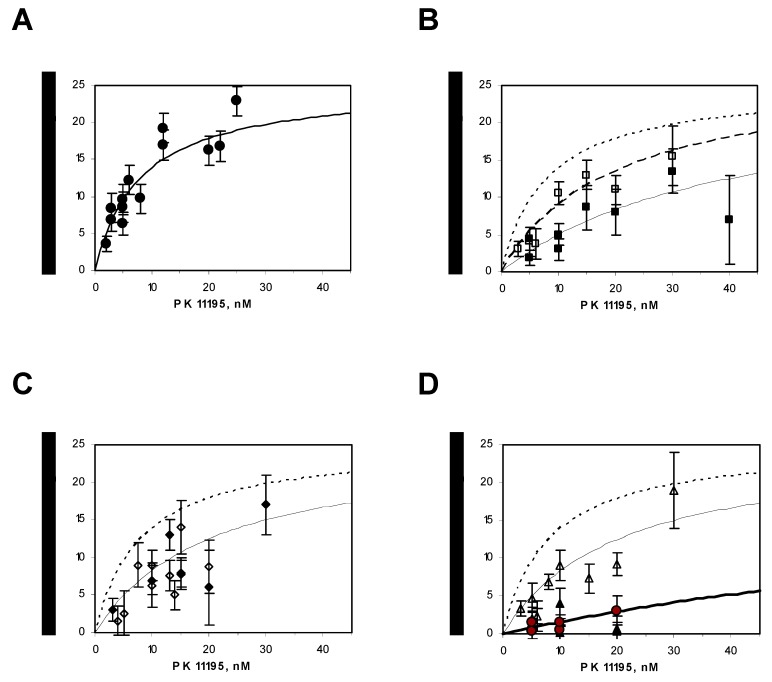
The saturation isotherms of [^3^H]-PK 11195 binding to reconstituted rec-mTSPO in proteoliposomes: (**A**) the wild type (WT) rec-mTSPO, (**B**) mutants Y34 (F and S opened and closed squares, respectively), (**C**) deletion mutants (Δ41–51 and Δ153–169 open and closed diamonds, respectively) and (**D**) mutant F100A, double mutants Y34F/F100A and Y34F/F99A (open and closed triangles and filled red circles, respectively). The lines represent the best-fit regression curves. The dashed line in panels (**B**–**D**) corresponds to that of WT presented in panel (**A**).

In our hands, the reconstitution of rec-mTSPO into proteoliposomes is the most efficient way to measure the PK 11195 high-affinity drug ligand binding constants using radioactive assays [23,28]. The saturation curves of PK 11195 binding to the WT and mutants of TSPO reconstituted in liposomes are presented in Figure 6. The data were fitted to obtain the affinity constant (K_d_) and stoichiometry (B_max_) (see Table 1). K_d_ varied from 8 nM for the WT to 150 nM for the double mutants (Y34F/F100A and Y34F/F99A), whereas all the stoichiometries obtained were very close and equal to 25–30 nmol of PK 11195 bound per mg of recombinant TSPO. This value of B_max_ is consistent with a saturation of 1 PK 11195 per TSPO, taking into account that (i) about 50% of the protein is facing the inside of the proteoliposome, a classical observation after membrane protein reconstitution in proteoliposomes [29], and (ii) a calculated maximal stoichiometry of approx. 50 nmol of PK 11195 bound per mg of recombinant TSPO (i.e., 1 mol of PK 11195 per mol of TSPO). Figure 6B shows that the point mutation of Y34 to serine (Y34S) led to a decrease in the apparent affinity compared with WT (Figure 6A), which is even more significant than when tyrosine is replaced by phenylalanine (Y34F). Both deletion mutants (Δ41–51 and Δ153–169) had the same affinity decrease as for Y34F. Interestingly, compared to the effect expected for the suppression of 10 residues, we observed only a mild decrease in affinity (Figure 6C [13,25]. The point mutation of the highly conserved F100 to alanine (F100A) alone induced a small decrease in affinity for PK 11195 binding, but the double mutant (Y34F/F100A) of the two highly conserved Y34 and F100 had a large effect (20-fold decrease) (Figure 6D). It has to be noted that the double mutant (Y34F/F99A), involving the less conserved F99, also had a large effect (20-fold decrease, Table 1), enforcing the hypothesis of hydrophobic interactions between PK 11195 and the aromatic residues driving the ligand to its binding pocket.

## 3. Discussion

The recent determination of the atomic structure of TSPO from different species produced valuable information about the PK 11195 ligand binding site [9,15,16,18]. However, the binding mechanism still remains elusive. The mammalian mTSPO in DPC micelles, studied by NMR, was stabilized upon ligand binding and displayed larger mobility and lower helix packing in the absence of PK 11195 than in its presence [9,22]. Conversely, bacterial *Bc*TSPO, studied by X-ray crystallography, displayed a similar atomic structure [16] with or without PK 11195. Therefore, it is not clear whether or how the ligand entered the binding pocket. Ligand binding is a dynamic process that involves several amino acids in helix packing and binding pocket formation. In its stabilized form, 61 NOEs between mTSPO and PK 11195 have been exploited for the NMR structure determination [9]. They involve 10 amino acids (A23, V26, L49, A50, I52, W107, A110, L114, A147 and L150, displayed in orange in Figure 3 (top), except for W107 shown in green), but an atomic structure analysis suggested the involvement of 5 additional amino acids (R46, W53, W95, D111 and W143), which are at a short distance from the bound PK 11195. Nevertheless, the transition between the free and bound structures involves probably even more residues and, in particular, those from cytosolic loops that are needed to reach the PK 11195 binding site located in between transmembrane helices. 

Surprisingly, the deletion of several segments of TSPO did not induce a complete loss of PK 11195 binding as previously described [12,13] (Figure S1). The first part of TM1 (N-terminus, i.e., residues 2–20 or 5–20) can be removed, leading only to a reduced affinity [12,13]. The first part of TM2 (Δ41–51) can also be removed without a great loss of the PK 11195 binding (herein and in Reference [13]). L49 and A50, which have been described as part of the binding pocket [9], can be deleted (Δ41–51) without large decreases in the affinity (herein and in Reference [13]). These two amino acids are then neither important to binding nor to the folding, in agreement with the conservation of the secondary structure that we observed by circular dichroism. Similarly, W107, A110 and L114 can be removed (Δ108–119) while still measuring the PK 11195 binding [13]. Further on, a study of the Δ141–152 mutant showed that the deletion of A147 and L150, two of the ligand-protein contact points, still kept almost 75% of the PK 11195 binding [13]. This observation suggests that these two amino acid contributors to the binding pocket are not essential. 

Instead, other amino acids are crucial contacts, since a single point mutation at V26T, as well as a deletion of 15–35 that includes V26, induces a complete loss of PK 11195 binding [12]. Δ15–35 lacks Y34 for which we showed the contribution of its aromatic part in PK 11195 binding, suggesting the necessity of aromatic residues in targeting PK 11195 to its hydrophobic pocket. This observation is also supported by the effect of mutating F100 in the second loop and is reinforced by the effect of the double mutation (Y34F/F100A) that had an even larger contribution than the single mutations of Y34 and F100. However, not all aromatic residues present in the region connecting the two first transmembrane helices are involved, since the mutation of W42 and W47 had no effect on the PK 11195 binding [12]. In addition, the mutation of charged residues in the first and second loops (R32G, K39G and R103A) had no effect on the PK 11195 binding [12], suggesting that electrostatic interactions are not required for the ligand–protein complex. Since the C-terminus (Δ153–169 or Δ158–169) decreased the binding of PK 11195 (herein and in References [12,13,25]), we can therefore propose a contribution from the three cytosolic-facing regions to the targeting of PK 11195 to its binding pocket. 

Human polymorphism rs6971, i.e., a natural mutation of A147T, is responsible for differences in the affinity of some TSPO ligands [7,30,31]. The presence of alanine instead of threonine induces a decrease of the affinity of one or two orders of magnitude for the PBR28 ligand [7], whereas it has no effect on the PK 11195 ligand [30]. The mouse TSPO mutation of A147T reveals that the binding pocket can adopt different “conformations” leading to slightly different structures [18]. Notably, V26 in TM1 is significantly more dynamic than the rest of the residues [31] and is placed opposite to A147 in TM5 in the PK 11195 binding cavity. Moreover, the TM1-TM2 loop conformation is divergent between the two polymorphs. The bacterial *Rs*TSPO A/T mutant reveals an "open" structure without the ligand rather than a “closed” structure for native TSPO [15]. Several differences are clearly observed between the two structures; TM2 was more tilted, and TM5 was less kinked for the A/T mutant, but more interestingly, the greatest change was observed in the loop connecting TM1 to TM2 (res29–40). Namely, in the “open” structure, the loop was not observed in the 3-D crystal, presumably due to a high flexibility. It is worth mentioning that the same loop was also poorly defined in the 2-D crystals of the same *Rs*TSPO by cryo-electron microscopy [26].

**Figure 7:**
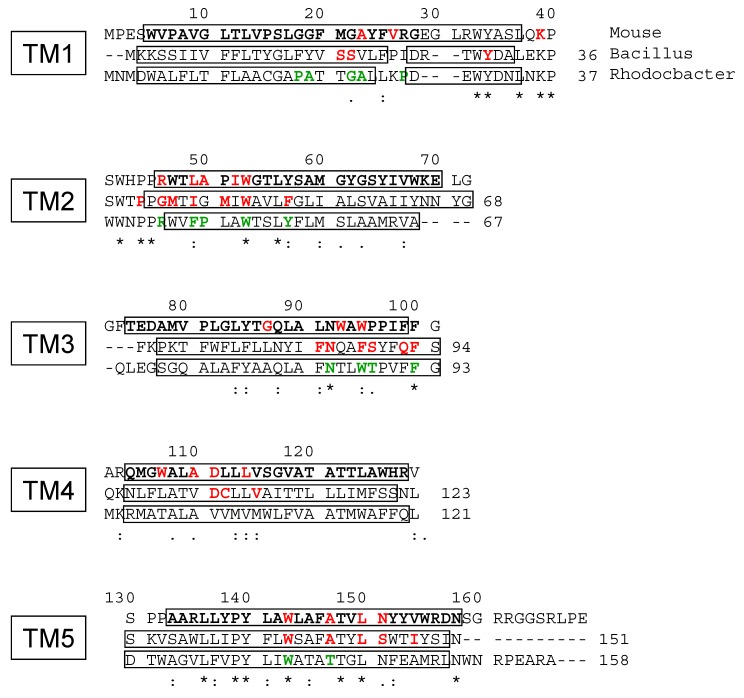
The sequence alignment of mouse, *Bacillus cereus* and *Rhodobacter sphaeroides* TSPO: The boxes depict the five transmembrane domains (labelled TM1 to TM5) as well as the short alpha helix in continuity with the first TM for *Bc*TSPO and *Rs*TSPO. The black bold amino acids are those involved in the transmembrane helices in the NMR atomic structure obtained from (mTSPO—PDB-2MGY). The amino acids involved in the PK 11195 binding pocket of the atomic structure obtained either from NMR (mTSPO—PDB-2MGY) or x-ray (*Bc*TSPO—PDB-4RYI) data are written in red bold characters. Those involved in the protoporphyrin IX (PPIX) binding pocket of the atomic structure from the x-ray data of *Rs*TSPO (PDB-4UC1) are in green.

Interestingly, the atomic structure of TSPO from *Rhodobacter sphaeroides* has been determined [15] with the protoporphyrin IX (PPIX) bound in the same pocket as PK 11195 in both mouse and *Bacillus cereus* TSPO. Amino acids interacting with both ligands are either conserved or homologous (Figure 7). Among them, F92 of *Rs*TSPO, highly conserved among TSPOs, is closer to the PPIX ligand than the equivalent F93 of *Bc*TSPO to the PK 11195 ligand. We showed that the mTSPO mutation of F100 to a short and nonaromatic residue such as alanine has a strong effect on the PK 11195 affinity when coupled to another mutation of an aromatic residue in the first loop. Our data suggesting a role for the loops in ligand recognition coupled with previous data indicating that the gating of the high-affinity ligand binding site is controlled by TM1 and TM5 tend to clarify the mechanism of ligand binding in TSPO. Whereas the C-terminus extending TM5 seems to participate to this process, the N-terminus located on the opposite side of the ligand binding pocket is not involved. Accordingly, the deletion of a long sequence from the N-terminus of *Arabidopsis thaliana* TSPO, *At*TSPO, has no effect on ligand binding [32]. Ligand binding might involve large changes in the structure of TSPO as observed in different NMR studies [9,10,18,22]. However, one may raise the question of the effect of detergents upon the structures determined by NMR when compared with structures determined by x-ray crystallography that show smaller conformational changes. On the one hand, PK 11195 bound did not have the same conformation by itself and is not superposable in the binding pocket of different atomic structures solved by NMR or x-ray crystallography. On the other hand, even if the three-dimensional fold of the 5 TM domains is conserved, the one-to-one superposition of individual TMs is different for the various TSPOs [9,15,16]. A recent paper suggests that high-resolution NMR studies obtained for some membrane proteins in DPC detergent correspond to nonfunctional states [33]. 

Several atomic structures of membrane proteins have been resolved by x-ray crystallography in a unique conformation, impairing our understanding of transport mechanisms, such as the case with the ATP/ADP Carrier, which the structure of has been determined only in the presence of the strong inhibitor carboxyatractyloside CATR [34]. Crystal formation may also constrain the conformation of cytosolic domains, as is the case of the first atomic structure of Ca-ATPase [35], which exhibited a domain separation that differs from the compact structure described by electron microscopy [36].

**Figure 8:**
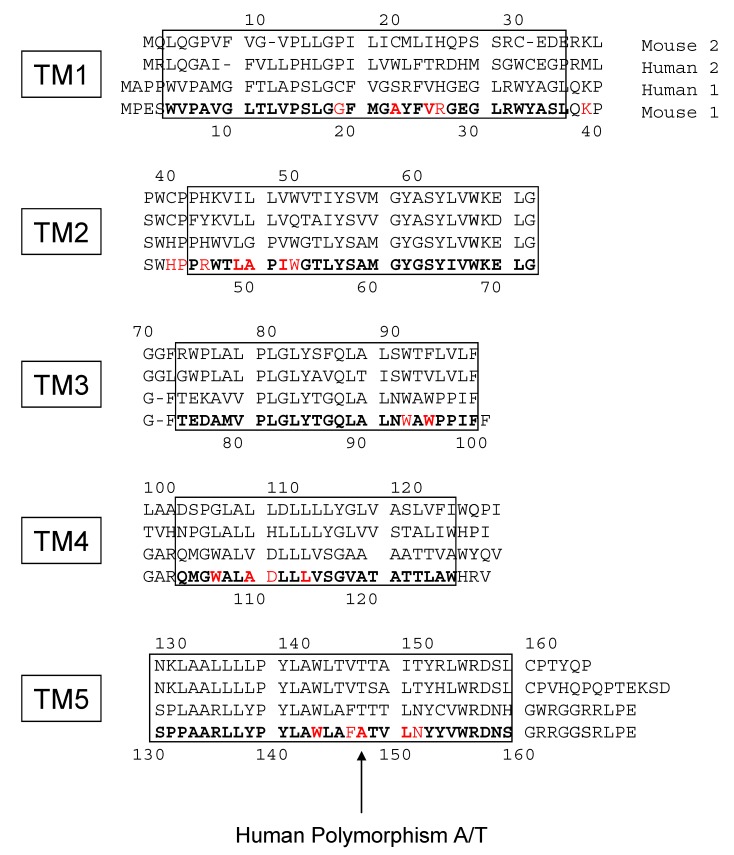
The sequence alignment and helix assignment of isoforms 1 and 2 of mouse and human TSPO: The boxes depict the five transmembrane helices (labelled TM1 to TM5). The numbers on top correspond to the isoform 2 positions, whereas the numbers at the bottom correspond to isoform 1. The amino acids involved in the PK 11195 binding pocket of the atomic structure obtained from NMR data are written in red bold characters, and those observed at a short distance (3 angstroms) are in normal red characters.

TSPO1 exhibits a high affinity for PK 11195, while its paralogous TSPO2 has been described as not binding to PK 11195 [17]. The question raised is the origin of such a difference. Looking at the binding of PK 11195 to mTSPO1 and *Bc*TSPO1, which are selective to the same (*R*) enantiomer of PK 11195, the bound ligand has different orientations of the rings and carbonyl group within the cavity, supporting a possible plasticity of the binding site. However, ligand binding requires the presence of various amino acids to make the pocket, and an analysis of the sequence alignment of the two proteins (Figure 8) reveals little amino acid conservation between TSPO1 and TSPO2. The crucial residues in the TM1 (A23 and V26), as well as in TM3 (W95) and TM4 (W107), that make up the binding site of PK 11195 in TSPO1 are not conserved in TSPO2. In particular, Y34 present in TSPO1 that we describe as important for targeting PK 11195 to the binding pocket is absent in TSPO2. While the two paralogous TSPOs do not share the same capacity to bind PK 11195, the site of cholesterol binding in the transmembrane region, one of TSPO’s hallmark functions, is highly conserved [37,38,39].

## 4. Materials and Methods

### 4.1. Expression and Purification of Recombinant Mouse TSPO

Mouse TSPO (mTSPO) was expressed in *E. coli* BL21 DE3, grown up in an LB medium and purified by His-binding to Ni-NTA chelation resin in the presence of 1% SDS according to published protocols [13,19,22,40]. The Protein purity was analyzed by SDS-PAGE (12% acrylamide) run on a Protean II system (BioRad, Marnes la coquette, France). The protein levels were quantified by UV spectra, using an absorption coefficient calculated from the amino acid sequence composition. When needed, SDS was exchanged with 0.2 % dodecylphosphocholine (DPC-h38 or DPC-d38) before protein elution from the Ni-NTA column (Qiagen, Les Ulis, France). Stable isotope labeled recombinant mTSPO was expressed in a M9 minimal medium complemented with 1 g/L [^15^N]-NH_4_Cl and 4 g/L glucose for fully labeled mTSPO, whereas selectively labeled mTSPO was obtained by adding a mixture of amino acids containing the desired [^15^N]-Lys to the M9 minimal medium [41,42]. 

### 4.2. NMR Experiments

The 2-D ^1^H–^15^N HSQC spectra were recorded at 30 °C on a Bruker 500 MHz spectrometer equipped with a cryogenic triple resonance probe with samples containing 0.1 mM mTSPO in 10 mM sodium phosphate (pH6) 90:10 H_2_O:D_2_O solutions containing 0.2% *w*/*w* DPC-d38 without or with PK 11195 at 10–20 molar ratios of PK 11195 over mTSPO. The HSQC spectra (pulse program hsqcetfp3gpsi, Bruker, Palaiseau, France) were collected with 2048 and 256 complex points in the direct and indirect dimension, respectively. The data were processed with Bruker Topspin 3.2.

### 4.3. Trypsin Digestion

Proteolysis was performed by mixing rec-mTSPO (0.2 mg/mL) solubilized in DPC (0.1%) and a phosphate buffer (10 mM at pH 7.0) with protease at a TSPO:Trypsin ratio of 30:1 (*w*/*w*). After various incubation times at 30 °C, the reaction was stopped by adding a Tosyl-L-lysyl-chloromethane hydrochloride (TLCK trypsin inhibitor at a ratio over trypsin of 2.5 (*w*/*w*), and the sample was placed on ice. PK 11195 from the stock solution (25 mM in ethanol) was added at chosen concentrations before trypsin addition. A control reaction without PK 11195 was performed to confirm that ethanol up to 4% had no effect on proteolysis. Tricine SDS-PAGE (16.5%) silver stained gels were used to follow proteolysis [43,44]. The apparent molecular weights of peptides generated by trypsin digestion were calculated using the relative migration distance to that of a set of molecular weight standards. 

### 4.4. Site-Directed Mutagenesis

Mutations were performed using the QuikChange Site-Directed Mutagenesis kit (Stratagene, France) according to published protocols [25]. In brief, miniprep pET-PBR plasmid double-strand DNA was used as a template. Synthetic oligonucleotide primer pairs containing a point mutation or deletion, each complementary to the opposing strand of the vector, were extended during temperature cycling by *pfu* DNA polymerase. The nicked vector DNAs containing the desired mutations were then transfected into *E. coli*. The mutations and deletions generated were confirmed by DNA sequencing. 

### 4.5. CD Spectroscopy

Far UV circular dichroism spectra were recorded on a Jobin-Yvon CD6 spectropolarimeter operated at room temperature according to published protocols [19,25,45]. Briefly, a detergent solubilized mTSPO sample (5 µM) in a 10 mM sodium phosphate buffer solution was placed in a 0.2 mm path length quartz cuvette (Hellma, Paris, France). The CD spectra were recorded in the 185 to 270 nm wavelength range with a 0.2 nm step resolution, 1 s signal averaging time and 1 nm bandwidth. The spectra were averaged over five scans, corrected for background and smoothed over 25 points. A consensus secondary structure content was estimated by spectral deconvolution using CONTINLL, CDSSTR and Selcon software and datasets of reference proteins SMP50 (37 soluble proteins and 13 membrane proteins) and SP37 (37 soluble proteins) [46], as well as CDFriend.

### 4.6. Fluorescence Spectroscopy 

The intrinsic fluorescence was recorded on a Biologic spectrophotometer operated at room temperature with excitation and emission wavelengths set at 290 nm and 340 nm, respectively. The fluorescence intensity of SDS-solubilized mTSPO (5 µM) placed in a 10 mM sodium phosphate buffer was recorded; DPC (1 mg/mL) was then added, and the change in fluorescence intensity was measured. 

### 4.7. Reconstitution of TSPO in Liposomes 

SDS-solubilized mTSPO was reincorporated in proteoliposomes by mixing with SDS solubilized lipids (dimyristoyl phosphatidyl choline/dimyristoyl phosphatidyl ethanolamine, 9:1) at a lipid to protein ratio of 5 (*w*/*w*). The detergent was removed using Bio-Beads SM2 according to published protocols [19,23,25,28,47]. Reconstitution was followed by combining the fluorescence and optical changes as previously described [19,28,47]. 

### 4.8. Radioligand-Binding Assays 

Proteoliposomes containing recombinant mTSPO were incubated with various concentrations of [^3^H]-PK 11195 (SA, 83.5 Ci/mmol, Perkin Elmer SAS, Courtaboeuf, France) in phosphate buffer saline (PBS). The bound ligand was quantified by liquid scintillation spectrometry after filtration on Whatman Filters GF/C (Sigma-Aldrich, Saint Quentin Fallavier, France) according to published protocols [20,25]. The K_d_ and B_max_ values were determined by fitting the saturation curves with the following equation *Ligand bound* = (*B*_max_ × *Ligand*)/(*K*_d_ + *Ligand*).

### 4.9. Molecular Graphics and Distances Calculation 

The molecular graphics and the distance between ligands and amino acids were drawn and measured, respectively, using Pymol software [48]. The following atomic structures of mTSPO, *Bc*TSPO and *Rs*TSPO were retrieved from the PDB: 2MGY and 2N02 for mTSPO; 4RYI, 4RYJ, 4RYM, 4RYN, 4RYO, 4RYQ and 4RYR for *Bc*TSPO; and 4UC1, 4UC2, 4UC3 and 5DUO for *Rs*TSPO. 

## 5. Conclusions

In summary, this study documents the involvement of the cytosolic loops of the mTSPO1 in the binding process of the PK 111195 ligand to its binding pocket using combined approaches of selective labelling of lysines, limited proteolysis by trypsin targeting arginines and lysines, point mutations of aromatic residues as well as deletion mutants, and radioligand binding. We showed that the interaction mechanism driving the ligand to its binding pocket probably involves interactions between PK 11195 and the aromatic residues of the mTSPO1, such as the tyrosines and phenylalanines of the cytosolic loops. These results shed new light on the ligand binding mechanism of TSPO, opening the way for the design of new drugs for diagnostics and therapeutics.

## Figures and Tables

**Table 1:** The mutant binding parameters: The affinities and stoichiometries were determined by fitting the saturation curves of bound radioactive [^3^H]-PK 11195 to rec-mTSPO reconstituted in proteoliposomes.

Mutation	K_d_ (nM)	B_max_ (nmol/mg)
WT	8 ± 3	25 ± 3
Y34S	40 ± 7	25 ± 5
Y34F	20 ± 3	27 ± 3
Δ41–50	20 ± 8	25 ± 8
ΔC-ter (153–169)	20 ± 5	30 ± 10
F100A	20 ± 5	25 ± 7
Y34F/F100A	150 ± 20	25 ± 10
Y34F/F99A	150 ± 20	25 ± 10

## Supplementary Materials

The following are available online at https://www.mdpi.com/1422-0067/20/6/1444/s1.

## Abbreviations

PK 11195	*N*-butan-2-yl-1-(2-chlorophenyl)-*N*-methylisoquinoline-3-carboxamide
TSPO	Translocator protein
mTSPO	mouse TSPO
*Bc*TSPO	*Bacillus cereus* TSPO
*Rs*TSPO	*Rhodobacter sphaeroides* TSPO
AtTSPO	*Arabidopsis thaliana* TSPO
HSQC	Heteronuclear Single Quantum Coherence
PPIX	Protoporphyrin IX
NMR	Nuclear Magnetic Resonance
DPC	Dodecylphosphocholine
CD	Circular Dichroism
TM	Transmembrane
WT	Wild Type
