# Supplementary material for: Characterization of the High-Affinity Drug Ligand Binding Site of Mouse Recombinant TSPO
Source: Int J Mol Sci. 2019 Mar 21;20(6):1444. doi: 10.3390/ijms20061444 (PMC6470738; doi:10.3390/ijms20061444)
Supplement: Supplementary file 1 [file ijms-20-01444-s001.pdf]

Figure S1

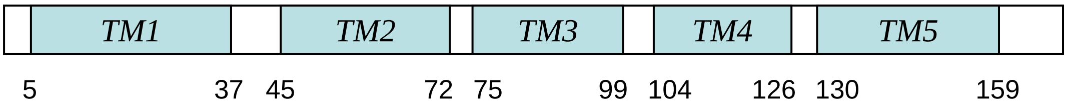

Yeast mitochondria	PK 11195 Kd (nM)	Bacterial protoplast	PK 11195 binding % of control
Wild-type	6 ± 3	Wild type	100
Δ2-20	25 ± 8	Δ5-20	~70
Δ15-35	>200	Δ41-51	~55
Δ158-159	10 ± 6	Δ108-119	~70
Δ157-169	9 ± 3	Δ120-133	~55
Δ156-169	10 ± 4	Δ141-152	~75
Δ2-20,158-169	>200	Δ153-169	~75
**Farges et al. (1994)** *Mol Pharm 46: 1160-1167.*		**Li et al. (1998)** *Endocrinology 139: 4991-4997.*	

Figure S1. (top) Diagram of the transmembrane helices of mTSPO from the atomic NMR structure [9, PDB ID-2MGY]. **(bottom)** Table summarizing the deletion mutants of mammalian TSPO presented in the literature.

Figure S2

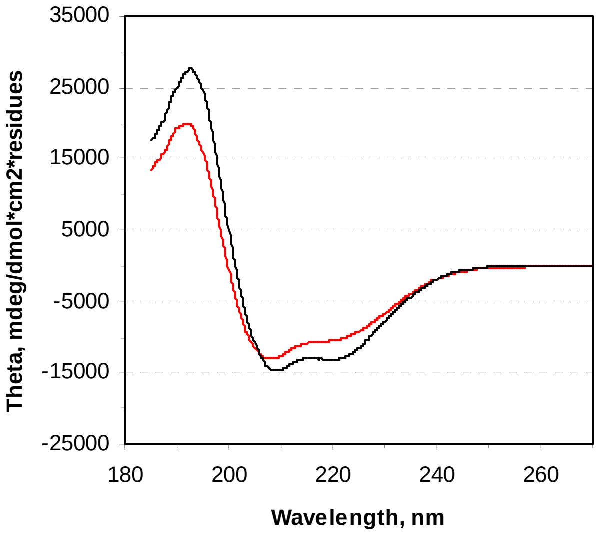

Figure S2. Circular dichroism spectra of rec-mTSPO. Spectra recorded in the presence of sodium dodecyl sulfate (SDS, red line) or in the presence of dodecylphosphocholine (DPC, black line) detergent**.**

Figure S3

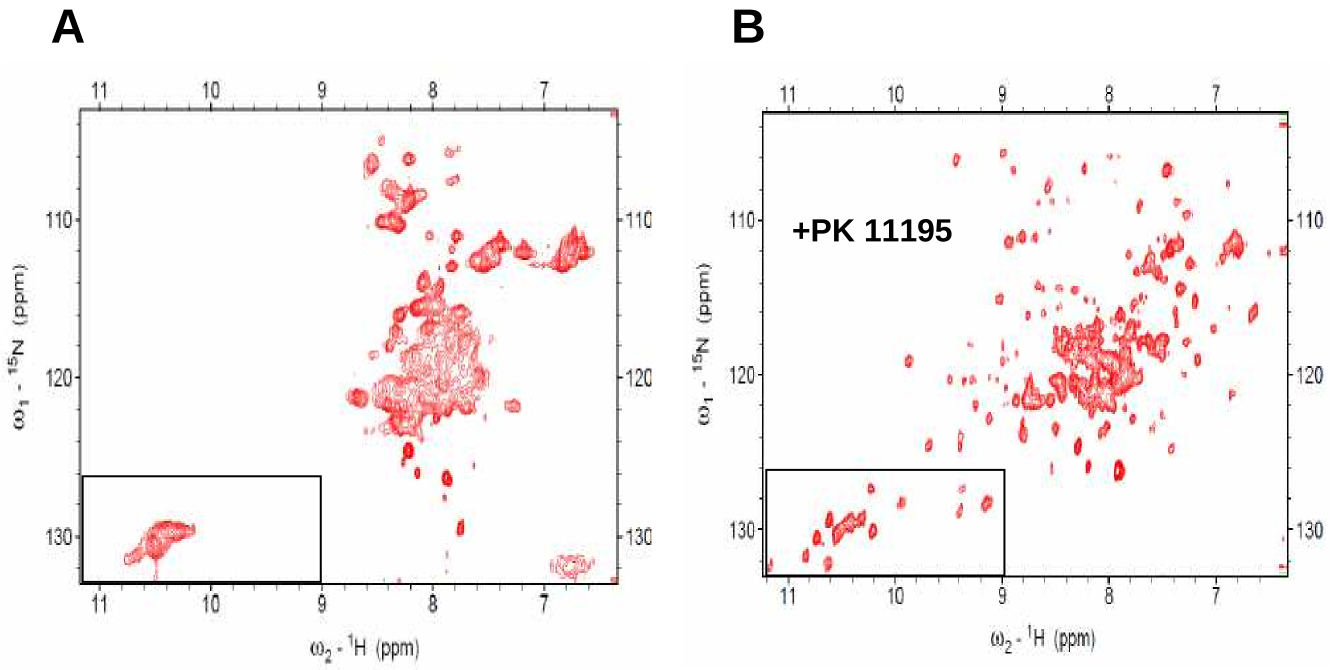

Figure S3. Ligand-induced stabilization of the rec-mTSPO structure in dodecylphosphocholine (DPC) detergent. 2D ^{1}H-^{15}N HSQC spectra of ^{15}N labelled rec-mTSPO in DPC micelles in the absence **(A)** and in the presence **(B)** of PK 11195. Boxes focus on NH indole resonance of tryptophans.
